# Molecular Role of HIV-1 Human Receptors (CCL5–CCR5 Axis) in neuroAIDS: A Systematic Review

**DOI:** 10.3390/microorganisms12040782

**Published:** 2024-04-12

**Authors:** Marcos Jessé Abrahão Silva, Rebecca Lobato Marinho, Yan Corrêa Rodrigues, Thiago Pinto Brasil, Pabllo Antonny Silva Dos Santos, Caroliny Soares Silva, Daniele Melo Sardinha, Karla Valéria Batista Lima, Luana Nepomuceno Gondim Costa Lima

**Affiliations:** 1 Postgraduate Program in Parasite Biology in the Amazon (PPGBPA), Evandro Chagas Institute (IEC), Ananindeua 67030-000, PA, Brazil; jesseabrahao10@gmail.com; 2 Institute of Biological and Health Sciences (ICB), University of Pará State (UEPA), Belém 66087-670, PA, Brazil; rebeccamarinho28@gmail.com (R.L.M.); antonnypabllo@gmail.com (P.A.S.D.S.); karolinysoares2303@gmail.com (C.S.S.); danielle-vianna20@hotmail.com (D.M.S.); 3 Faculty of Medicine, Federal University of Ceará (UFC), Fortaleza 60441-750, CE, Brazil; theutlys@gmail.com; 4 Bacteriology and Mycology Section (SABMI), Evandro Chagas Institute (IEC), Ananindeua 67030-000, PA, Brazil; karlalima@iec.gov.br (K.V.B.L.); luanalima@iec.gov.br (L.N.G.C.L.)

**Keywords:** HIV, neurological manifestations, CCR5, CCL5, immunity

## Abstract

Chronic HIV-1 infection can cause neurological illness, also known as HIV-associated neurocognitive disorders (HAND). The elevated level of pro-inflammatory cytokines and chemokines, such as C-C Chemokine Ligand 5 (CCL5/RANTES), is one of the ways of causing HIV-1-mediated neuroinflammation. C-C Chemokine Receptor 5 (CCR5) is the main coreceptor for viral entry into host cells and for mediating induction of CCL5/RANTES. CCR5 and CCL5 are part of a correlated axis of immune pathways used for effective protection against the HIV-1 virus. The purpose of this paper was to review the literary knowledge about the immunopathological relationship between this immune complex and neuroAIDS. A systematic review of the literature was conducted based on the selection and search of articles, available in English, Spanish, or Portuguese in the time frame of 1990–2022, of primary and secondary types in the PUBMED, Science Direct, SciELO, and LILACS databases through descriptors (MeSH) together with “AND”: “CCR5”; “CCL5”; “neurological manifestations”; or “HIV”. The methodological quality of the articles was assessed using the JBI Checklists and the PRISMA 2020 writing guidelines were followed. A total of 36 articles were included in the final composition of the review. The main cells of the CNS affected by neuroAIDS are: neurons; microglia; astrocytes; and oligodendrocytes. Molecular devices and their associations with cellular injuries have been described from the entry of the virus into the host’s CNS cell to the generation of mental disorders. Furthermore, divergent results were found about the levels of CCL5/RANTES secretion and the generation of immunopathogenesis, while all condensed research for CCR5 indicated that elevation of this receptor causes more neurodegenerative manifestations. Therefore, new therapeutic and interventional strategies can be conditioned on the immunological direction proposed in this review for the disease.

## 1. Introduction

The human immunodeficiency virus (HIV) can impair the immune system by targeting the CD4+ T lymphocyte cells, which causes the acquired immune deficiency syndrome (AIDS) [1]. Type 1 HIV (HIV-1) has been shown to produce a wide range of neurological diseases and penetrates the central nervous system (CNS) shortly after infection [2,3]. Patients with HIV-1 infection have a significant frequency of HIV-associated neurocognitive disorders (HAND) [4,5].

Currently, combined antiretroviral therapy (cART) is the standard of care for HIV-positive patients who are following the right treatment regimens. Although it has improved the quality of life of people with HIV, cART is unable to eliminate the virus’ latent reservoir [6]. As a result, HIV/AIDS is now considered a chronic condition requiring continuing treatment rather than a fatal disease. At least half of patients receiving cART exhibit HIV-associated neurocognitive disorders (HAND), which have been linked to HIV-1 infection severity and its replication in the CNS, despite substantial viral load reduction [7].

Primary HIV neurological disease occurs when HIV is both necessary and sufficient to cause the illness [5]. Besides that, primary HIV-related CNS disease is driven by the viral load and inflammation in the brain at the time the CD4+ count is drawn, and it will function largely independently of other variables like HIV disease stage, viral load, or dyslipidemia [8]. The occurrence of a specific neurological illness in an HIV-positive individual is the result of a series of circumstances that are influenced by the features of HIV itself, such as the genetic makeup of the host, immunological features, and interactions with the environment (including therapy) [9,10].

HIV can interact with additional pathogens to cause tumors and opportunistic infections (OI), secondary or opportunistic neurological disease, and treatment-related neurological disease (like immune reconstitution inflammatory syndrome, or IRIS). Some examples of HIV-infected cells include microglia, monocytes, macrophages, astrocytes, and the choroid plexus. Infections are expected to impact neurons and oligodendrocytes seldom, if at all, and “indirect” processes are assumed to oversee the bulk of damage. However, this is still up for discussion [11].

Since C-C chemokine receptor 5 (CCR5) is the main coreceptor that allows HIV-1 infection when coupled with the viral glycoprotein 120 (gp120), it has been identified as a possible target for anti-HIV therapies [12]. Immune cells (such as T cells), smooth muscle endothelial cells, epithelial cells, CNS cells, even parenchymal cells, and other cells exhibit CCR5, also known as CD195, a G-protein-coupled receptor (GPCR) whose expression is regulated by cAMP response element-binding (CREB)-18 protein [13]. When it comes to the processes that start HIV-1 infections, virions attach to the surface of target cells to start the adsorption phase. This is accomplished by a high affinity contact between the CD4+ T lymphocyte being the primary receptor and the extracellular domain of the viral glycoprotein gp120. Following this glycoprotein’s attachment to the CD4+ T receptor, the cell membrane undergoes structural modifications that allow for interaction with co-receptors like CCR5, which facilitates the fusing of the viral envelope with the host cell membrane and, ultimately, facilitates viral entrance [14].

In this sense, the polarization of cells with immunological expression and the activation of lymphocytes can be both mediated by C-C Motif Chemokine Ligand 5 (CCL5/regulated on activation, normal T-cell expressed and secreted—RANTES), which binds to G protein receptors [15]. CCL5/RANTES has the greatest affinity with CCR5, but it may also connect to CCR1, CCR3, and CCR4. Most inflammatory cells, mainly T cells and macrophages, are capable of producing CCL5/RANTES [16]. As a critical regulator of T-cell migration to inflammatory sites, CCL5/RANTES is a chemokine that directs T-cell migration to damaged or infected regions [17]. Additionally, research suggests that CCL5/RANTES controls T-cell differentiation through type 1 of T helper (Th1) cells recruitment [18].

CCL5/RANTES may disrupt the interaction between the HIV-1 Env gp120 and the receptor, which is necessary for viral entry, by competitively binding to CCR5 [19]. This can prevent HIV-1 cells from entering and replicating in those cells. Furthermore, CCL5/RANTES might cause the internalization of the bound receptor, which in turn reduces the expression of CCR5 on the cell surface [20]. Chronic HIV-1 infection can cause neurological illness, often known as neuroAIDS. The elevated level of pro-inflammatory cytokines and chemokines, such as CCL5/RANTES, is one of the ways of causing HIV-1-mediated neuroinflammation, leading to HIV-1-mediated neurotoxicity (neuroAIDS) [21]. Research evidence demonstrated that HAND has a positive relationship with immunological activation and had a more wide spectrum of cognitive abnormalities that may overlap with other brain disorders [22]. Then, this work had the purpose of systemizing the immunopathological relationship between the CCL5–CCR5 immune axis and neuroAIDS progression.

## 2. Material and Methods

### 2.1. Study Design

This systematic review was performed in compliance with the Preferred Reporting Items for Systematic Reviews and Meta-Analyses (PRISMA) 2020 statement [23]. The PICO technique was employed to formulate the guiding question, corresponding the anagram for population (P), intervention (I), comparison (C), and outcome (O). In this regard, it was produced from the following: P—patients with HAND; I—assess CCL5–CCR5 immune response; C—CCL5–CCR5 via and HAND biological effects; O—neuroAIDS advancement in CNS [24]. It led to the formulation of the guiding question: “What immunopathological components within the CCL5–CCR5 axis are involved in the progression of HAND in CNS?”.

### 2.2. Search Strategy

The identification and selection of articles were performed in the databases Science Direct (https://www.sciencedirect.com/; accessed on 20 November 2023), the National Library of Medicine National Institutes of Health of the USA—PUBMED (https://pubmed.ncbi.nlm.nih.gov/; accessed on 21 November 2023), Scientific Electronic Library Online—SciELO (https://www.scielo.br/; accessed on 26 November 2023), and Latin American and Caribbean Literature in Health Sciences—LILACS (https://lilacs.bvsalud.org/; accessed on 26 November 2023), using the descriptors: “CCL5”, “CCR5”, “neurological manifestations” and “HIV”, together with the Boolean operator “AND”. The period included in the analysis was between January 1990 and December 2022. The information was gathered on 21 August 2023. This search strategy only considered English, Portuguese, and Spanish languages.

If a study satisfied all three criteria, it was deemed to be eligible: (i) dealt specifically with HIV-1; (ii) reported information on the biological effects of CCR5 and/or CCL5 on the progression of neuroAIDS; and (iii) had data on the CNS. Before selecting the data to be extracted, the study titles and abstracts were reviewed.

### 2.3. Data Extraction

All information deemed pertinent, including discrepancies and ambiguities discovered in the publications, was separately extracted by the two authors (MJAS and CSS), with the assistance of a third author (RLM) in circumstances where the selection of the data was discordant. The data that were extracted comprised the author’s name, publication year, title, method, database, and outcomes.

Data gathering and a review of the publications’ methodological quality were performed at the same time. Joanna Briggs Institute (JBI) checklists were used to conduct a methodological quality assessment [25]. Pre-defined criteria from the literature were used to include two articles through their methodologies [26,27]. A third author (TPB) discussed any discrepancies regarding the study’s inclusion between the two researchers’ analyses (MJAS and CSS).

## 3. Results

### 3.1. Literature Search

**Figure 1:**
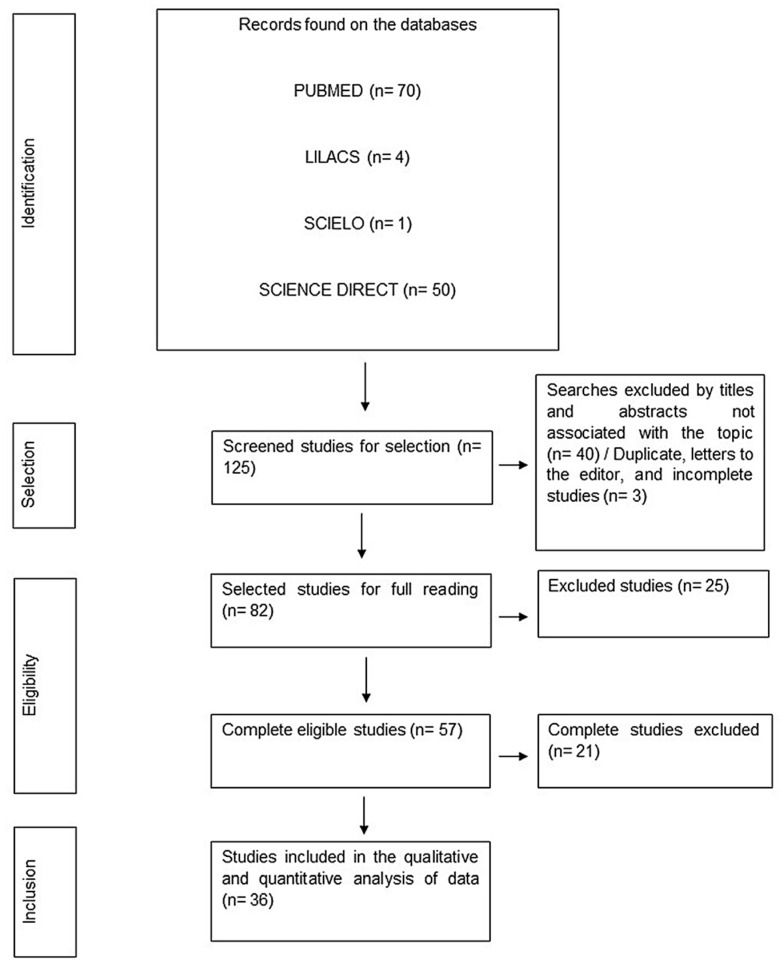
PRISMA flowchart of the stages of selection of the articles within this systematic review.

The search in four databases identified 125 articles, with only 3 duplicated or incomplete works. Based on titles and abstracts that were unrelated to the topic’s focus, 40 publications were subsequently eliminated from consideration. Consequently, 82 articles were chosen for thorough reading, of which 46 were dropped for deviating from the subject. A total of 36 studies fit this criterion (Figure 1), and they were included.

### 3.2. Characteristics of the Studies

Table 1 describes the characteristics of the 36 studies included in this systematic review. The majority came from the PUBMED database (n = 36; 100%), of American origin (n = 22; 61.11%) and of the experimental type (n = 18; 50%). All presented high methodological quality within the score acquired in the JBI Checklist for each specific type of study included.

### 3.3. Effects of HIV-1 Infection on Cellular and Molecular Pathways from the Perspective of the CCL5–CCR5 Immune Axis

Immune response against HIV-1 infection in the brain initiates with action of APC cells, such as macrophages, dendritic cells, and CD4+ T cells in the bloodstream, which recognize pathogen-associated molecular patterns (PAMPs) and pathogen-associated molecular damage (DAMPs) of the virus through virus recognition receptors patterns (PRRs), such as toll-like receptors (TLRs) to initiate an innate immune response through factors, such as inflammatory mediators [63]. In this instance, CCR5 acts to fuse the viral membrane with that of the host cell and introduce viral particles into the cell to begin the viral replication process [64].

The process of neurotoxicity related to HAND was related to a series of viral pathogenic elements inside the affected cell that guide and drive the progression of the infection, whether directly or indirectly [65]. The neurotoxicity of HIV-1 and HIV-1 proteins, such as gp120, Tat, and Nef, is what causes the direct effects of CNS infection with HIV-1 [28,33,40,41,57,60]. In contrast, toxic mediators like quinolinic acid and arachidonic acid metabolites, as well as pro-inflammatory cytokines, are secreted by microglia or astrocytes that are either infected with HIV-1 or exposed to HIV-1 proteins, causing indirect neurotoxicity [38]. Astrocytes make up around 70% of the brain, and these cells are prone to non-productive HIV-1 infection as well as low levels of productive infection [39,41,49]. Since these cells oversee preserving brain homeostasis, they are crucial in mediating the neurotoxic consequences of CNS HIV-1 infection. As a result, there is the phosphorylation of molecules related to the activation of the NF-kB pathway, which release, in the nucleus, molecules related to the subsequent increased release of CCL5/RANTES, correlated with the increase in neuroinflammation and cellular damages in the brain [62]. The main cells of the CNS affected by neuroAIDS, as described in the literary scene, are: neurons; microglia; astrocytes; and oligodendrocytes [32,40,42,51,52,53,56,60,66]. These tissue injuries cause clinical problems in affected individuals [67].

Neurocognitive deficits including diminished attention/concentration, psychomotor speed, memory, learning, information processing, and executive function are some of the neurocognitive symptoms of this process that are clinically manifest [68]. Also present are motor slowness, incoordination, and tremor, which can develop into paraparesis, debilitating weakness, spasticity, and extrapyramidal movement disorders [69]. Apathy and impatience are only a couple of the possible behavioral side effects. It is also possible to have psychomotor slowness, which is linked to frontal-striatal system injury [70]. Clinically, this process can range in severity from asymptomatic neurocognitive impairment (ANI) to moderate neurocognitive disorder (MND) to full-blown HIV-associated dementia (HAD) [71]. Since the initial diagnosis of AIDS dementia complex (ADC), HIV-associated cognitive-motor complex (HIVCMC), and most recently HIV-associated neurocognitive disorders (HAND), a variety of clinical nosologies have been used to describe this process [72].

**Figure 2:**
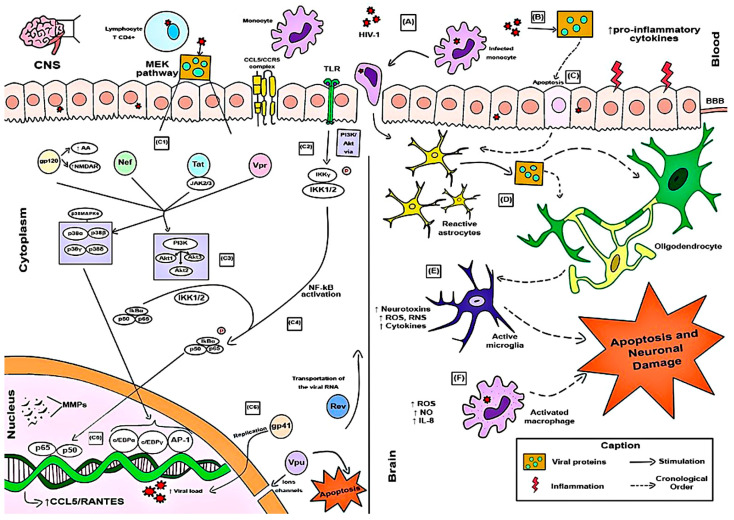
CCL5–CCR5 axis induction by HIV-1 infection and HIV-1 neurotoxicity effects in molecular and cellular levels of the central nervous system (CNS). Their stages are indicated in chronological order of events by letters (A–F). It describes the stages of immune processes in the human body from viral entry into the CNS cells and the processes of adhesion and recognition by APCs (A), viral replication process releases viral proteins that exacerbate the inflammatory process (B), and the activation of cell signaling pathways (C): in the cellular cytoplasm, viral proteins act on joint pathways for secretion of co-stimulatory molecules of this inflammation (C1); toll-like receptors (TLRs) activate other protein pathways that induce phosphorylation of molecules related to the activation of the NF-kB pathway (C2–C4); with subsequent increased release of CCL5/RANTES and matrix metalloproteinases (MMPs) associated with neuroinflammation (C5); other viral proteins act by increasing the viral load and generating an inflammatory cycle (C6). Ultimately, they generate reactive astrocytes, affected oligodendrocytes, and neurons, and ROS, RNS, inflammatory mediators and neurotoxins determine apoptosis, inflammation of neurons, and other CNS cells (in the absence of cell death), and damage to neuronal cells (D–F).

Figure 2 describes the main findings of the articles included in this review regarding the process of HAND from the perspective of the CCL5–CCR5 pathway, considering the viral pathological effects since viral entry into the host’s CNS cells. It demonstrates how devices induced by the pathogen molecularly deregulate the immune response of the affected cell to the point of affecting the pathway and leading to a worse mental prognosis in humans with cell death and neural damage.

## 4. Discussion

Research using postmortem tissue from AIDS patients, in situ hybridization [73], and immunohistochemical methods [74] have been used to show the presence of HIV-1 in the brain. Brain is a key location for viral infection and replication, which can happen in the early stages of HIV-1 sickness [75]. It has been established that the release of CCL5/RANTES plays a critical role in modifying HIV-1 replication in mononuclear phagocytes in the blood and lung, and that the expression of CCR5 on T cell surfaces determines vulnerability to HIV/AIDS through variation in its activation [30].

Key immune activity regulators of the host can be represented by chemokines and their accompanied chemokine receptors [51,76]. Homeostatic and inflammatory are the two kinds of main families of the chemokines [77]. In immune homeostasis, homeostatic chemokines are primarily expressed in lymphoid organs and control leukocyte trafficking to these locations, whereas inflammatory chemokines (such as CCL5/RANTES) are induced at infected/damaged tissues and draw leukocytes to areas that have experienced an inflammatory insult [17].

The CCL5–CCR5 axis prevents tissue macrophages from dying from virus-induced cell death, hence providing antiapoptotic signals for macrophage survival during infection [35]. CCL5–CCR5 association is a key regulator of endothelial progenitor cells’ homing during wound healing, suggesting that CCL5 may be significant as a general B cell coactivator [14]. Additionally, the interaction between CCL5 and its derivatives with CCR5 is thought to be a possible treatment axis for HIV-1 since the gp120 protein of HIV-1 binds to chemokine receptors CCR5 [15] or CXCR4 [12] as a major phase of HIV-1 entrance into the host cell.

Chemokine receptors play crucial functions in the attraction and placement of immune cells within tissues and have been linked to a variety of CNS inflammatory illnesses [55]. Correlative research links the CCR5–chemokine axis to a variety of illnesses, including cerebral malaria, Rasmussen encephalitis, progressive multifocal leukoencephalopathy-associated immune reconstitution inflammatory syndrome, and HAND issues [58]. In addition, it has been demonstrated that CCL5/RANTES contributes to the development of Parkinson Disease and multiple sclerosis, i.e., other neurodegenerative illnesses [78,79]. In this context, this work sought to review the AIDS neuropathogenesis induced by the CCL5–CCR5 immune pathway and build an illustration in the article for didactic visualization of related immune and biological processes.

Many viral proteins can cause direct harm to neurons. Numerous neuronal cell surface receptors, including the N-methyl-D-aspartate receptors (NMDAR), the low-density lipoprotein receptor-related protein (LRP), the chemokine receptors CCR5 and CXCR4, and the dopamine transporter, play a major role in mediating this sensitivity [79,80]. The most prevalent neurotransmitter in the brain, glutamate, is responsible for facilitating the transmission of excitatory signals by binding to NMDAR and opening cation-specific channels in cell membranes, which permit the entry of sodium, calcium, and potassium ions while blocking the exit of other ions [81]. LRP is involved in the movement of cholesterol inside neurons, as well as signaling and preventing apoptosis in these cells [82].

Dopamine receptors and transporters are less common than NMDAR, but they are still significant because they are present in regions of the brain that are vulnerable to HIV-1 infection, such as the striatum and substantia nigra, which are involved in executive function and the behavioral reward system [50]. Additionally, dopamine transporter expression is lower in the striatum of HIV patients with cognitive deficits [11]. The two main viral proteins, Tat and gp120, interact with the receptors to harm neurons. Both monomeric and oligomeric gp120 have neurotoxic properties [48,60], and transgenic mice producing gp120 show a variety of neuronal and glial alterations reflecting abnormalities in the brains of HIV-1-infected people [34].

The expression of genes including CCR5, CXCR4, CD4, and CypA, as well as factors that inhibit viral replication like TRIM5 and APOBEC3G, take on greater significance in this research area [83]. They produce human proteins (such as the chemokines) that are directly related to the host’s immune response against the pathogen [84]. Additionally, it is known that genetic polymorphisms in the HIV-1 coreceptors (such as in *CCR5*) and their ligands (like RANTES/CCL5) affect several aspects in immune response, including the severity of the infection [85,86].

Recent meta-analysis by Silva et al. (2023) conducted analysis of genetic variants, more precisely three functional single nucleotide polymorphisms (SNPs) of CCL5/RANTES and demonstrated that reduced expression of this gene decreases the level of CCL5 protein production and increases risk of HIV-1 progressing to AIDS [87]. Previous meta-analyses and systematic reviews carried out to evaluate SNPs and insertions and deletions (Indels) in the CCR5 gene (such as the CCR5-delta32 variant) also claimed that increased expression of this viral coreceptor in host cells and increases the susceptibility and severity of AIDS [85,88]. The study by Tamamis and Floudas (2016) with bioinformatics methods predicted the potential benefits of the CCL5–CCR5 immune axis and provides insights into the blocking mechanism of HIV-1 by CCL5/RANTES [20].

Although studies have already been carried out relating variants of these genes to the worse prognosis of HIV-1 disease, to date, no epidemiological association study between genetic polymorphisms of CCL5/RANTES and/or CCR5 has specifically sought relationships for cohorts of individuals. with neuroAIDS. Thus, the research conducted so far regarding both gene and protein expression suggests that the elevation of these factors conditioned to CCL5/RANTES appears to mitigate the clinical evolution of general AIDS, and vice versa [89].

According to the results found in this present study, divergent data were found regarding the expression levels of the chemokine CCL5/RANTES and its role in neuroinflammation, while all research that analyzed CCR5 in patients with neurological manifestations associated with AIDS, there were positive associations between increased receptor synthesis and greater brain damage, so as reported by previous research [90,91].

In this case, unlike what has already been proposed about the associations between the regulation of gene expression and, consequently, the production of the CCL5/RANTES protein related to the susceptibility and/or progression of HIV-1 infection, high levels of CCL5/RANTES may be related to both pro- and anti-inflammatory roles against apoptosis in neuroAIDS. However, it was also noted in this review that all studies that reported a direct relationship in their results between increased CCL5 and greater neurotoxicity in these patients had a serum concentration of CCL5/RANTES > 20 nM (156 ng/mL).

Therefore, it is suggested the existence of a relative threshold between CCL5/RANTES expression levels in the CNS of the infected individual for their effective immune response against the pathogen, through the CCL5–CCR5 axis, as many studies in this review also concluded a beneficial organic role in the evolution of the neurological clinical picture related to the elevation of the levels of this chemokine (all at a concentration of CCL5/RANTES ≤ 20 nM) in the blood of individuals with AIDS.

In this sense, inflammation is a defense device necessary for an immune response required in disease, while an excessive amount is deleterious. An example of these cases are severe cases of COVID-19, in which these individuals develop, at an advanced stage of the disease, a molecular positive feedback condition that exacerbates the inflammatory condition in the body, which can generate multiple organ dysfunction. However, this inflammatory response triggered in the initial immune response to SARS-CoV-2 in initial cases of infection is crucial to avoid a worsening prognosis and inflammatory progression to the point of causing organic disorders, through inflammatory manifestations, such as a cytokine storm [92].

In this context, this article makes a pioneering hypothesis about the possible cause of the divergence already reported through observations of similar data from the included studies. If this hypothesis is confirmed by new primary studies on the disease, one of the possible causes for the greater cerebral pathogenesis in these cases could be hypercytokinemia correlated with a hyperinflammatory condition.

Regarding CCR5, desensitization, internalization, and recycling or receptor degradation all play a role in the control of its expression. According to Oppermann (2004), these pathways are started by either homologous or heterologous phosphorylation of the receptor/ligand complex by protein kinase C (PKC) or G protein-coupled receptor kinases [93]. Therefore, several studies have already reported the effectiveness of CCR5 antagonist drugs in proposing measures against HIV-1 infection, corroborating the findings of this review for associated with factors, such as neurological complications [94]. Besides that, although neuronal death is associated with HAND (which is usually thought of as severe cognitive impairment), the more relevant importance of inflammation in the era of ART is in the minor cognitive impairment that is seen in up to 25% of those infected with HIV. In these cases, there is no sign of neuronal death and the cognitive impairment is thought to be a direct effect of inflammation resulting in neuron dysfunction [4,95].

In addition to CCR5 antagonists, CCR5-ligand chemokine RANTES serves as an appropriate molecular building block for the development of effective HIV drugs. In the search for new HIV-1 entry inhibitors, RANTES-engineering techniques have been extremely valuable through the formulation of potential CCL5/RANTES derivatives [96]. The ongoing development of chosen molecular leads is being driven by a “mixed” strategy that combines structure-guided design with empirical testing. HIV microbicides based on CCL5/RANTES might be given exogenously and generated in a gel carrier to sustain protective concentrations for a long time [97].

As an alternative, bacterial systems that can produce recombinant CCL5/RANTES derivatives endogenously following colonization of the female genital tract (live microbicides) might be created. When safe and efficient RANTES derivatives that may prevent HIV-1 transmission to intraepithelial CD4+ T cells and mononuclear phagocytic cells in the vaginal canal become available, the field of microbicides may benefit the most [98]. Therefore, this present study can provide support for therapeutic applications and new forms of treatment, although primary studies are still needed to confirm the information described about the role of CCL5/RANTES levels in neuroAIDS [99,100]. 

Some previous reviews [101,102] have already been conducted regarding the association between this immune axis and neuropathogenesis caused by AIDS disease. However, this review is a pioneer in presenting a scheme in image format relating immunopathology to the CCL5–CCR5 complex. The study limitations identified were: (1) each study’s respective definition of HIV-1 infection based on case identification; (2) each study’s respective definition of neuroAIDS severity based on case identification; (3) the methodology applied in each study; (4) the need for different HIV-1 variants to evaluate data from these included studies; (5) the methodology adopted for this systematic review. Future epidemiological studies with cohorts of diverse populations aimed at identifying new molecular pathways involved and characterizing the CCL5–CCR5 complex in these individuals are essential for defining new panoramas of health strategies [103].

## 5. Conclusions

Host factors that mediate the neurotoxic damage set on by the CNS being subjected to HIV-1-infected cells, HIV-1, or certain HIV-1 proteins. Different diseases have varying degrees of severity due to different neurotoxic pathways, such as the CCL5–CCR5 axis. Astrocytes, neurons, and microglia all express the chemokine receptor CCR5. CCR5 directly affects the progression of the neurological condition. It was shown that CCL5 can have distinct impacts on viral infectivity and neurotoxicity when it comes to neuroAIDS.

This review also concludes that greater attention should be given to subsequent studies aiming at therapeutic improvement, through the observation and association between clinical-epidemiological factors of individuals with neuroAIDS and the molecular concentrations of CCL5/RANTES and CCR5 in the blood, aiming to create a new molecular biomarker for monitoring and monitoring these patients.

Then, it was suggested here that there are several ways to precisely counteract the neurotoxicity of viral and host proteins, as well as possible neuroprotective elements that may be strengthened to lessen the detrimental effects of HIV-1 on the brain. Direct neuronal death, abnormal control of essential neuronal support cells, and dendritic arbor loss are the main factors that cause HAND. Therefore, new therapeutic and interventional strategies can be conditioned on the immunological direction proposed in this review for the disease.

## Figures and Tables

**Table 1:** Data analyzed of the articles included in this review.

N°	Title	Author and Year of Publication/Database/(JBI Score)	Methodology	Results
1	CCR5 mediates HIV-1 Tat-induced neuroinflammation and influences morphine tolerance, dependence, and reward.	Gonek et al. (2018) [28]/PUBMED/JBI (9/9)	Experimental study	Tat and opioids that act on the mu opioid receptor (MORs) seem to promote CCR5 and CCR2 signaling, causing neuroinflammation and striatal neurotoxicity to increase. The reduced therapeutic effectiveness and enhanced rewarding characteristics of morphine that we have discovered herein may be caused by these effects.
2	Differential expression of the alternatively spliced OPRM1 isoform μ-opioid receptor-1K in HIV-infected individuals.	Dever et al. (2014) [29]/PUBMED/JBI (10/11)	Cohort	HIVE was linked to enhanced expression of MCP-1, MCP-2, and CCL5/RANTES.
3	Nonproductive human immunodeficiency virus type 1 infection of human fetal astrocytes: independence from CD4 and major chemokine receptors.	Sabri et al. (1999) [30]/PUBMED and Science Direct/JBI (8/9)	Experimental study	On the cell surface of human fetal astrocytes, there is no indication of any of the key HIV-1 coreceptors, such as CXCR4, CCR5, CCR3, and CCR2b, or the CD4 molecule. However, RT-PCR was able to identify the mRNA transcripts for CXCR4, CCR5, Bonzo/STRL33/TYMSTR, and APJ. Furthermore, results demonstrate that primary HIV-1 isolates can infect astrocytes by a mechanism unrelated to CD4 or important chemokine receptors (such CCL5/RANTES). Additionally, astrocytes have the potential to be latent HIV-1 carriers and, upon activation, may play a role in the transmission of the infection to nearby cells like macrophages or microglia.
4	Encephalopathy in AIDS—increased formation of beta-chemokines in monocytes after HIV-1 virus infection: mechanisms of CNS involvement.	Mayer; Schmidtmayerová (1997) [31]/PUBMED/JBI (10/11)	Cohort	When HIV illness is advanced, dementia is linked to an increased viral load in the brain. The most plausible pathogenetic mechanism causing brain dysfunction and damage is neurotoxicity linked to the activity of macrophages and microglial cells that are HIV-infected. Our research has shown that HIV infection of macrophages significantly increases the production of the chemokines MIP-1-alpha, MIP-1-beta, and RANTES/CCL5 (subgroup C-C). Due to their chemoattractant and activating qualities, these substances may contribute to leukocytosis and inflammation, which in turn raises the population of HIV-susceptible cells, facilitates their infection, and ultimately increases intrathecal virus spread.
5	Microglia express CCR5, CXCR4, and CCR3, but of these, CCR5 is the principal coreceptor for human immunodeficiency virus type 1 dementia isolates.	Albright et al. (1999) [32]/PUBMED/JBI (9/9)	Experimental study	In HIV dementia (HIVD) patients, CCR5 was expressed at greater levels by microglia than CCR3 or CXCR4. It revealed that only CCR5 and CXCR4 of these three chemokine receptors could transduce a signal in microglia in response to their respective ligands, MIP-1 and SDF-1. Since the anti-CCR5 antibody 2D7 was able to significantly reduce microglial infection by both wild-type and single-round luciferase pseudotype reporter viruses, we also discovered that CCR5 is the main coreceptor used for infection of human adult microglia by the HIV type 1 dementia isolates HIV-1DS-br, HIV-1RC-br, and HIV-1YU-2.
6	A Bivalent Ligand Targeting the Putative Mu Opioid Receptor and Chemokine Receptor CCR5 Heterodimers: Binding Affinity versus Functional Activities.	Yuan et al. (2013) [33]/PUBMED/JBI (9/9)	Experimental study	While the CCR5 and MOR may coexist as heterodimers in human astrocytes, the CCR5 receptor may exist as a monomer or form homodimers in monoclonal receptor-expressed assays. The CCR5 binding pocket in the heterodimer may accommodate the bivalent ligand, preferably to cause a blockage of viral entry.
7	Genetic knockouts suggest a critical role for HIV co-receptors in models of HIV gp120-induced brain injury.	Maung et al. (2012) [34]/PUBMED/JBI (11/11)	Review	Apoptosis and neuronal damage are brought on by HIV-1 proteins like gp120, which drive macrophages to generate neurotoxins. Additionally, the two main HIV co-receptors, the chemokine receptors CCR5 and CXCR4, have a wide range of physiological uses and are expressed in cells other than immune cells, such as those in the brain. Transgenic mice with HIV gp120 expression in the brain resemble NeuroAIDS brains in various pathogenic ways. Among CCR5-expressing and CCR5-deficient animals, there does not appear to be a difference in peripheral production of the chemokines CCL5/RANTES, CCL3/MIP-1, or CCL4/MIP-1ß. However, in contrast to wild-type controls, CCR5 Knockout (KO) mice exhibit improved humoral or cell-mediated immune responses, depending on the antigen trigger.
8	Autophagy is increased in postmortem brains of persons with HIV-1-associated encephalitis.	Zhou; Masliah; Spector (2011) [35]/PUBMED/JBI (9/10)	Case-control	In neural cells exposed to either CXCR4- or CCR5-tropic HIV-1 gp120, levels of autophagic proteins and autophagosomes were elevated. The etiology of neuroAIDS is significantly influenced by the dysregulation of autophagy during HIV infection.
9	Immunoregulation of a CB2 receptor agonist in a murine model of neuroAIDS.	Gorantla et al. (2012) [36]/PUBMED/JBI (9/9)	Experimental study	In neural cells exposed to either CXCR4- or CCR5-tropic HIV-1 gp120, levels of autophagic proteins and autophagosomes were elevated. CCR5 expression on CD4+ cells was considerably decreased with Gp1a therapy in both immunodeficient mice reconstituted with human peripheral blood lymphocytes (hu-PBL) and HIVE mice, according to flow cytometric studies. Between the treatment and control groups, the levels of CCR5 expression on CD8+ cells were not substantially different.
10	Chemokine receptor-5 (CCR5) is a receptor for the HIV entry inhibitor peptide T (DAPTA).	Polianova; Ruscetti; Ruff (2005) [37]/PUBMED/JBI (9/9)	Experimental study	According to their results, DAPTA (a CCR5 antagonist) reduces the binding of the viral envelope to CCR5 in R5-tropic HIV isolates. This inhibition most likely occurs through competitive binding of peptide T (DAPTA), a non-toxic experimental antiviral entry inhibitor derived from HIV-1, to CCR5. The degradation of gp120’s interaction to the chemokine receptor CCR5 is what gives DAPTA its specific antiviral effect for HIV-1 R5-tropic viruses.
11	Viral and cellular factors underlying neuropathogenesis in HIV associated neurocognitive disorders (HAND).	Rao; Ruiz; Prasad (2014) [38]/PUBMED/JBI (11/11)	Review	Although neurons do not support HIV-1 infection or replication, they do express several cell-surface receptors (such as CCR5, CXCR4, NMDAR, etc.) that make them vulnerable to insults from viral proteins (such as Tat, gp120), inflammatory cytokines (such as TNF-alpha, IL-1), and small metabolites (such as nitric oxide, arachidonic acid, etc.) secreted by immune cells in the brain. The N-methyl-D-aspartate receptors (NMDAR), the low-density lipoprotein receptor-related protein (LRP), the chemokine receptors CCR5 and CXCR4, and the dopamine transporter are the key mediators of this susceptibility. HIV-1 gp120 can bind to either CCR5 or CXCR4 and cause neuroblastoma cell death. Gp120 can directly bind to CCR5 or CXCR4, starting a signaling cascade driven by p38-MAPK that causes neuronal death. The natural ligands of both CCR5 (such as CCL5, CCL3) and CXCR4 (such as CXCL12) were discovered to be neuroprotective against gp120 neurotoxicity.
12	HIV-1 Envelope Protein Gp120 Up-Regulates CCL5 Production in Astrocytes Which Can Be Circumvented by Inhibitors of NF-ΚB Pathway.	Shah et al. (2011) [39]/PUBMED/JBI (10/11)	Cohort	The function of CCL5 in promoting CCL2 production, which can facilitate leukocytes infected with HIV-1 migrating through the BBB. The gp120-induced mRNA expression of CCL5 is time-dependent. Increased CCL5 levels may encourage monocyte migration through the BBB, aggravating neuroinflammatory illness. Additionally, CCL5 has a protective function in the setting of HIV-1 infection at doses of 10 nanomoles (nM) (equal to 78.76 ng/mL), promoting neuronal survival under pro-apoptotic conditions. However, the maximal levels of CCL5 induction by gp120 in the current investigation were only 3 ng/mL, preventing the concentration from being protective. CCL5 rise mediated by Gp120 can only be understood as responsible for a neuroinflammatory response. A particular NF-kB inhibitor and siRNA might partially stop the elevated CCL5 synthesis.
13	Vpr- and Nef-Dependent Induction of RANTES/CCL5 in Microglial Cells.	Si et al. (2002) [40]/PUBMED/JBI (9/9)	Experimental study	HIVD patients’ CSF fluids have been discovered to have elevated levels of RANTES/CCL5. These brains exhibit both HIV-1 and inflammatory mediators, which can promote RANTES/CCL5 synthesis. In astrocytes, microglia, and macrophages, CCL5/RANTES can be activated by cytokines and viral substances (recombinant HIV-1 proteins). CCL5 is produced with a delayed kinetics associated with increased viral replication, and necessitates HIV-1 infection. CCL5 secretion is unaffected by X4-viruses, and is not inhibited by cytokine antagonists or antibodies. The viral accessory protein Vpr, in addition to Nef, is required for the synthesis of RANTES/CCL5, indicating a unique function for Vpr in chemokine induction in primary macrophage-type cells. Additionally, the p38 MAP kinase lowers chemokine expression in microglia.
14	HIV-1 Nef Induces CCL5 Production in Astrocytes through P38-MAPK and PI3K/Akt Pathway and Utilizes NF-KB, CEBP and AP-1 Transcription Factors.	Liu et al. (2014) [41]/PUBMED/JBI (7/9)	Experimental study	Nef astrocytes exhibit a substantial induction of CCL5. CCL5 expression by HIV-1 proteins has shown that NF-kB plays a significant role. Through the p38-MAPK and PI3K/Akt pathways, HIV-1 Nef stimulates the synthesis of CCL5 in astrocytes while making use of the transcription factors NF-kB, CEBP, and AP-1.
15	Characterization of HIV-1 Infection in Microglia-Containing Human Cerebral Organoids.	Gumbs et al. (2022) [42]/PUBMED/JBI (9/9)	Experimental study	The CCR5 co-receptor supports productive HIV infection in cerebral organoids and isolated organoid-derived microglia (oMG), both of which have been infected with replication-competent HIV reporter viruses. The co-expression of markers unique to microglia and the HIV CD4 and CCR5 receptors was necessary for HIV infection susceptibility.
16	Cellular localization of the chemokine receptor CCR5. Correlation to cellular targets of HIV-1 infection.	Rottman et al. (1997) [43]/PUBMED/JBI (9/9)	Experimental study	CCR5 is expressed by astrocytes, microglia, and neurons. When there is chronic inflammation, there are more CCR5-immunoreactive cells present, and this immunoreactivity is positively correlated with the severity of the inflammation as measured by histopathology.
17	Interaction of the CC-Chemokine RANTES with Glycosaminoglycans Activates a p44/p42 Mitogen-Activated Protein Kinase-Dependent Signaling Pathway and Enhances Human Immunodeficiency Virus Type 1 Infectivity.	Chang et al. (2002) [44]/PUBMED and Science Direct/JBI (11/11)	Cohort	It implies that RANTES can activate signaling pathways through an alternate receptor or receptors that are GAG dependent, but which are presumably not known, particular GPCR chemokine receptors, at both low, physiologically appropriate concentrations and at greater, potentially supraphysiological concentrations. At a post entry stage of the viral life cycle, the subsequent activation of MAPK at high RANTES concentrations can improve the efficacy of HIV-1 replication. Protein tyrosine kinases (PTK)- and MAPK-dependent signaling pathway is activated by the interaction of CCL5/RANTES with cell surface, glycosaminoglycans (GAGs).
18	Rantes distribution and cellular localization in the brain of HIV-infected patients.	Vago et al. (2001) [45]/PUBMED/JBI (10/10)	Case-control	RANTES was highly expressed in instances of inflamed brain lesions (22/24 HIV-positive patients and 2/7 HIV-negative patients). Microglial cells and lymphocytes were found to be positive in both diffuse and nodular areas. Only individuals who tested positive for HIV had positive astrocytes. RANTES was never negative in multinucleated giant cells.
19	CCR5 blockade for neuroinflammatory diseases--beyond control of HIV.	Martin-Blondel et al. (2016) [46]/PUBMED/JBI (10/11)	Review	HIV infection of CNS-resident cells is facilitated by CCR5, which is expressed in the CNS of HIV patients. Because CCR5 encourages the migration of immune cells into the CNS, it may have a role in the neurocognitive dysfunction linked to HIV. In experimental animals, the maraviroc monotherapy (a CCR5 antagonist drug) decreased microglial activation, major pro-inflammatory mediator expression, and levels of indicators of neuronal damage. The addition of maraviroc medication to antiretroviral therapy may lead to improvements in neurocognitive state, according to small trials.
20	Increased CCR5 Affinity and Reduced CCR5/CD4 Dependence of a Neurovirulent Primary Human Immunodeficiency Virus Type 1 Isolate.	Gorry et al. (2002) [47]/PUBMED/JBI (11/11)	Cohort	In some CNS illness patients, HIV-1 variants with elevated CCR5 affinity and decreased dependency on CCR5/CD4 might be present. These viruses would result from adaptive evolution aimed at infecting target cells that express relatively little CD4. Our research further indicates that a subgroup of neurotropic R5-viruses with higher CCR5 affinity and neurotropic R5X4-viruses may be involved in CNS neurodegenerative pathways.
21	Env gp120 sequence analysis of HIV type 1 strains from diverse areas of the brain shows preponderance of CCR5 usage.	Shah et al. (2006) [48]/Science Direct/JBI (9/10)	Case-control	CCR5, CXCR4, and CCR3 are expressed by microglia in the brain. It was postulated that HIV-1 neurodegenerative symptoms may be caused by enhanced CCR5 affinity, a pathogenic viral phenotype. HIV-1 from peripheral nerves is mostly CCR5 dependent and causes neuroinflammation and neuronal death.
22	Infection of Fetal Human Astrocytes by M-Tropic HIV Is Not Inhibited by Prostaglandin-Mediated CCR5 Downregulation.	Abdel-Haq; Hao; Lyman (1999) [49]/PUBMED/JBI (10/11)	Cohort	The expression of CCR5 can be reduced in human astrocytes by prostaglandins (PGE1, PGE2, and PGF1α), however they are unable to prevent infection by a monotropic strain of HIV. This implies that other coreceptors, which have not yet been discovered, are involved in the CNS infection of HIV and the development of neuroAIDS.
23	CCL2/Monocyte Chemoattractant Protein-1 Mediates Enhanced Transmigration of Human Immunodeficiency Virus (HIV)-Infected Leukocytes across the Blood–Brain Barrier: A Potential Mechanism of HIV–CNS Invasion and NeuroAIDS.	Eugenin et al. (2006) [50]/PUBMED/JBI (8/9)	Experimental study	Few monocytes infected with the X4-virus or lymphocytes infected with HIV contribute to increased monocyte transmigration in any manner. However, when R5-viruses are utilized, the extent of monocyte transmigration is much greater, demonstrating that viral tropism is critical in promoting monocyte transmigration and subsequent BBB rupture. On HIV-infected leukocytes, CXCR3 and CCR5 are retained or expressed at higher levels.
24	HIV-1 coreceptors CCR5 and CXCR4 both mediate neuronal cell death but CCR5 paradoxically can also contribute to protection.	Kaul et al. (2005) [51]/PUBMED/JBI (9/9)	Experimental study	RANTES and MIP-1β, which activate an Akt-dependent signaling pathway, provide neuroprotection. Contrarily, gp120, which prefers CCR5, causes neuronal death. Depending on the precise situation, the properties of the ligand, and the ensuing signaling cascade, CCR5 can either benefit or hurt the neurological system. Heterologous desensitization of CXCR4 receptors on neurons underlies the neuroprotective effects of CCR5-binding MIP-1β and RANTES.
25	A novel bivalent HIV-1 entry inhibitor reveals fundamental differences in CCR5-μ-opioid receptor interactions between human astroglia and microglia.	El-Hage et al. (2013) [52]/PUBMED/JBI (9/9)	Experimental study	It is believed that gp120/gp41-CCR5 interactions will occur when CD4 is present on microglia but not astrocytes. In addition to CCR5, it was shown that human microglia express MOR at extremely low relative levels as compared to astrocytes. When MOR is activated, CCR5 is upregulated in human host cells, which promotes HIV-1 infection and replication.
26	CCR5 knockout prevents neuronal injury and behavioral impairment induced in a transgenic mouse model by a CXCR4-using HIV-1 glycoprotein 120.	Maung et al. (2014)/PUBMED [53]/JBI (7/9)	Experimental study	Innate immune response elements might be triggered by gp120 without the need for the CCR5 or neuronal damage. CCL2, CCL5, and CXCL10 are a few of the up-regulated factors that have been linked to HIV neuropathogenesis. Neurotoxicity in HIVD is CCR5 dependent.
27	Co-receptor signaling in the pathogenesis of neuroHIV.	Nickoloff-Bybel et al. (2021) [54]/PUBMED/JBI (11/11)	Review	Patients with HIVE have some viral proteins in their brains, including gp120, which may have a neurotoxic effect. It was discovered a connection between CSF viral levels and neurological impairment, further pointing to a connection between viral persistence and neuroHIV. Interactions between host CNS cells and the infected cells that make up a persistent reservoir of HIV in the brain are a major contributor to neuropathogenesis in people on cART. By binding to both CXCR4 and CCR5 on neurons, the viral proteins that are shed, such as gp120, actively cause neurotoxicity. By secreting neuroinflammatory mediators including inflammatory cytokines and chemokines, infected and activated macrophages and microglia induce neuronal damage. The interaction of HIV virions with CXCR4 and CCR5 can activate other myeloid populations and trigger signaling pathways that might directly cause neuroinflammation.
28	The chemokine receptor CCR5 in the central nervous system.	Sorce et al. (2011) [55]/Science Direct/JBI (10/11)	Review	Through preventing interactions between the HIV gp-120 and the host CD4+ T cell and macrophage CCR5 receptors, CCR5 antagonists reduce HIV adherence to target cells in the host.
29	CCR3 and CCR5 are co-receptors for HIV-1 infection of microglia.	He et al. (1997) [56]/PUBMED/JBI (11/11)	Cohort	CCR3 and CCR5 are both utilized to allow viral entrance in microglial cells. Subsets of HIV-1 isolates that are M-tropic primarily utilize CCR3, with CCR5 serving as a secondary receptor.
30	A central role for glial CCR5 in directing the neuropathological interactions of HIV-1 Tat and opiates.	Kim et al. (2018) [57]/PUBMED/JBI (10/11)	Cohort	Striatum neurons were shielded against co-exposure to HIV-1 Tat and morphine by loss of glial but not neuronal CCR5. Loss of CCR5 prevented the interaction between Tat and morphine and restored the toxic effects of Tat in the cultures while morphine was still present. In mechanisms unrelated to its crucial involvement in HIV infection, CCR5 is implicated, at least in part through changing the equilibrium between proBDNF and mBDNF levels. The varied morphine/opioid toxicity in the presence or absence of CCR5 also raises questions about the complicated interactions between CCR5 and MOR, which may entail heterologous interactions.
31	The role of CCR5 in HIV-associated neurocognitive disorders.	Riviere-Cazaux et al. (2022) [58]/PUBMED/JBI (11/11)	Review	In addition to impairing axonal regeneration following neuronal injury, CCR5 activation can result in neuronal cAMP response element-binding protein (CREB) and mitogen-activated protein kinase (MAPK) inactivation. The gp120 V3 domain’s direct association with CCR5 may also impair synaptic plasticity, which would inhibit memory without triggering neuroinflammation.
32	Association of Chemokine (C-C Motif) Receptor 5 and Ligand 5 with Recovery from Major Depressive Disorder and Related Neurocognitive Impairment.	Bauer et al. (2021) [59]/PUBMED/JBI (10/11)	Cohort	Even before starting therapy, CCR5 and CCL5 levels were much lower in the responder group than in the nonresponder group. Regarding neurocognitive impairment in MDD patients, it was discovered that after five weeks of treatment, a greater misperception of the emotion “anger” was linked to a more pronounced change in CCR5, and that a faster perception of the emotion “disgust” was linked to a greater decrease in CCL5 over the same period.
33	HIV-1 Tat-Mediated Induction of CCL5 in Astrocytes Involves NF-κB, AP-1, C/EBPα and C/EBPγ Transcription Factors and JAK, PI3K/Akt and p38 MAPK Signaling Pathways.	Nookala et al. (2013) [60]/PUBMED/JBI (9/9)	Experimental study	Peak mRNA and protein levels for CCL5 expression were seen in Tat at 1 h and 48 h after transfection, respectively, indicating a time-dependent increase in the expression of the gene. CCL5 was up-regulated by AP-1, C/EBP-alpha, and C/EBP-gamma.
34	CCR5 is a suppressor for cortical plasticity and hippocampal learning and memory.	Zhou et al. (2016) [61]/PUBMED/JBI (8/9)	Experimental study	Since CCR5 is a potent memory and plasticity suppressor, HIV-related cognitive impairments may be exacerbated by viral proteins that overactivate CCR5.
35	Impact of Plasma IP-10/CXCL10 and RANTES/CCL5 Levels on Neurocognitive Function in HIV Treatment-Naive Patients.	Ruhanya et al. (2021) [62]/PUBMED/JBI (11/11)	Cohort	The most reliable indicators of the global deficit score (GDS) associated with neurocognitive impairment were IP-10 and RANTES. Both cytokines had an inverse relationship with CD4+ T cell counts and were positively connected with lymphocyte proviral load and plasma viral load. With the greatest closeness, IP-10 and RANTES formed their own cluster.
36	New Challenges of HIV-1 Infection: How HIV-1 Attacks and Resides in the Central Nervous System.	Rojas-Celis et al. (2019) [5]/PUBMED/JBI (11/11)	Review	Since macrophages are CD4+ cells and express the CXCR4 and CCR5 coreceptors, they are as vulnerable to HIV-1 infection as microglia are. However, the most common coreceptor in macrophages is CCR5, as data show that CXCR4-mediated entry of HIV-1 particles into macrophages does not confer any potential for infection. CCR5 is more strongly linked than CCR3 to viral entrance and the subsequent onset of dementia. It is feasible to find HIV-1 as infectious virions, viral proteins, and even nucleic acids in cultured astrocytes even though they do not exhibit CD4 or CCR5. Despite not expressing CD4 or CCR5, oligodendrocytes do express CXCR4. CXCR4, CCR5, and CCR3 are surface-expressed receptors in adult neurons. Inflammatory cytokines, ROS, and RNS that surround neurons provide an environment that is pro-inflammatory and reactive, which harms the neurons.

## Data Availability

The original contributions presented in the study are included in the article. Further inquiries can be directed to the corresponding author.
